# Impact of PBP4 Alterations on β-Lactam Resistance and Ceftobiprole Non-Susceptibility Among *Enterococcus faecalis* Clinical Isolates

**DOI:** 10.3389/fcimb.2021.816657

**Published:** 2022-01-20

**Authors:** Lorenzo M. Lazzaro, Marta Cassisi, Stefania Stefani, Floriana Campanile

**Affiliations:** Section of Microbiology, Department of Biomedical and Biotechnological Sciences (BIOMETEC), Microbiologia Medica Molecolare e Antibiotico Resistenza (MMARLab), University of Catania, Catania, Italy

**Keywords:** *Enterococcus faecalis*, time-kill curve assays, PBP4, ceftobiprole, *pbp*4 gene expression

## Abstract

Penicillin-resistance among *Enterococcus faecalis* clinical isolates has been recently associated with overexpression or aminoacidic substitutions in low-affinity PBP4. Ceftobiprole (BPR), a new-generation cephalosporin, is a therapeutic option against *E. faecalis.* Here, we present evidence that *pbp*4 gene sequence alterations may influence the expression level of the gene and ceftobiprole binding to PBP4 in *E. faecalis* clinical isolates showing remarkable MDR-phenotypes, and how this could interfere with BPR *in vitro* antibacterial and bactericidal activity. Seven *E. faecalis* strains from bloodstream infections were analyzed for their antibiotic and β-lactam resistance. BPR bactericidal activity was assessed by time-kill analysis; *pbp*4 genes were sequenced and *pbp*4 relative expression levels of transcription were performed by RT-qPCR. Five penicillin-resistant ampicillin-susceptible (PRAS) isolates were detected, 4 of which were also BPR non-susceptible (BPR-NS). In the time-kill experiments, BPR exposure resulted in a potent bactericidal activity (3-5 log_10_ reduction) at the different concentrations tested. *pbp*4 gene sequence analysis revealed some mutations that may account for the changes in PBP4 affinity and MIC increase in the 4 BPR-NS strains (MICs 4-16 mg/L): the deletion of an adenine (*del*A) in the promoter region in all PRAS/BPR-NS strains; 12 different amino acid substitutions, 7 of which were next to the PBP catalytic-sites. The most significant were: T418A, located 6 amino acids (aa) upstream of the catalytic-serine included in the ^424^STFK^427^ *motif* I; L475Q, 7 aa upstream of the ^482^SDN^484^ *motif* II; V606A and the novel Y605H, 13/14 aa upstream of the ^619^KTGT^622^ *motif* III. Taken together, our data showed that elevated BPR MICs were attributable to increased transcription of *pbp*4 - associated with a single upstream adenine deletion and PBP4 alterations in the catalytic-site *motifs* – which might interfere with the formation of the BPR/PBP4 complex. *pbp*4 molecular alterations may account for the changes in PBP4 affinity and MIC increase, without affecting BPR *cidal* activity. Indeed, our *in vitro* dynamic analysis by time-kill assays showed that BPR exerted a bactericidal activity against *E. faecalis* clinical isolates, despite their MDR phenotypes.

## Introduction

Enterococci are the third most commonly isolated nosocomial pathogens, accounting for 12% of all hospital infections (Hollenbeck and Rice, 2012). The clinical importance of the genus *Enterococcus* is closely related to antibiotic resistance, which contributes to the risk of infection.

Enterococci have high-level resistance to most cephalosporins and all semi-synthetic penicillins.

Among the species of greatest clinical interest, *E. faecalis* is intrinsically resistant to most β-lactams and only susceptible to a limited group of penicillins, such as ampicillin, penicillin and piperacillin (Arias and Murray, 2012; Kristich et al., 2014a).

Ampicillin resistance has been rarely reported in *E. faecalis*, as this did not represent a clinical and therapeutic challenge. Until recently, it was assumed that ampicillin- susceptible *E. faecalis* was also susceptible to penicillin, but *E. faecalis* clinical isolates have been exhibiting increasing levels of resistance to penicillin, due to the emergence of Penicillin-Resistant Ampicillin-Susceptible (PRAS) isolates, eliminating β-lactams as a treatment option. This uncommon phenotype has been reported in various hospitals worldwide but its real epidemiological impact is still unknown (Metzidie et al., 2006; Guardabassi et al., 2010; Tan et al., 2014; Cabrera et al., 2020; Conceição et al., 2020; Gawryszewska et al., 2021).

Reduced susceptibility to β-lactams in *E. faecalis* is attributable to two main mechanisms: the first is the production of β-lactamases, rarely described among *E. faecalis* strains (Rice and Murray, 1995; Sarti et al., 2012; Schell et al., 2020), while the second is the over-production of a single low-affinity class B penicillin-binding protein (PBP), named PBP4. The PBP4 active site in the Trans-Peptidase (TPase) domain encompasses three conserved *motifs*: the ^424^STFK^427^ *motif* I, containing the catalytic serine; the ^482^SDN^484^ *motif* II, involved in the protonation of the β-lactam leaving group; and the ^619^KTGT^622^ *motif* III, which facilitates substrate binding and defines the oxyanion hole (Ghuysen, 1991; Djorić et al., 2020). Accumulation of point mutations in the penicillin-binding module of PBP4 has been associated with a decreased affinity for β-lactams (Ono et al., 2005; Zapun et al., 2008; Infante et al., 2016; Moon et al., 2018; Rice et al., 2018; Gawryszewska et al., 2021).

The pandemic led to an alarming increase of *E. faecalis* isolated from patients with COVID-19 under mechanical ventilation and ICU-acquired enterococcals BSI (Giacobbe et al., 2021; Posteraro et al., 2021), also worsened by their increasing multi-resistance to all therapeutic options. *E. faecalis* pathogens play a crucial role in determining the severity of the clinical conditions, critically influencing the patients’ outcome, and represent a serious threat in infection therapy (Kim et al., 2019).

Among 5^th^ generation cephalosporins, ceftobiprole exerts superior *in vitro* antibacterial and bactericidal activity also against vancomycin-resistant and β-lactamase producing strains, due to its high affinity for PBPs (Mendes et al., 2016; Hamilton et al., 2017; Campanile et al., 2019).

The aims of this study were: 1) to investigate the *in vitro* antibacterial and bactericidal activity of BPR alone against *E. faecalis* clinical isolates belonging to selected antibiotic-resistance classes; 2) to analyze the occurrence of *pbp*4 mutations and verify their role in influencing the activity of β-lactams and, specifically, of BPR; 3) to compare the *pbp*4 expression levels in all *E. faecalis* clinical isolates with reduced susceptibility to beta-lactams and PBP4 alterations, with the aim of evaluating which of these alterations may be involved in non-susceptibility and BPR *cidal* activity, and how.

## Materials and Methods

### Strains

Seven *E. faecalis* clinical strains, isolated from bloodstream infections (BSI) in Italian hospitals, were selected for their antibiotic-resistance behaviors from a larger collection of twenty-two isolates already characterized (Campanile, et al. 31st ECCMID 2021, P 2004). They belonged to the major MDR phenotypes (PRAS, BPR-NS, VRE, HLAR); two beta-lactam-susceptible *E. faecalis* isolates were also selected for comparison.

*E. faecalis* OG1RF, deposited in the American Type Culture Collection (ATCC) under ATCC 47077, deriving from *E. faecalis* OG1 by selection for resistance to rifampin and fusidic acid (Bourgogne et al., 2008), was used as control in molecular studies. *E. faecalis* ATCC 29212 was used as control for antibiotic-susceptibility tests (EUCAST, 2021).

### Antimicrobial Susceptibility Testing

Ceftobiprole was provided by Basilea Pharmaceutica International Ltd. (Basel, Switzerland); ceftaroline, linezolid and tigecycline by Pfizer Inc. (New York, NY, USA); daptomycin by Novartis (Basel, Switzerland). Penicillin, ampicillin, amoxicillin, imipenem, vancomycin, teicoplanin, gentamicin and streptomycin were purchased commercially (Sigma Chemical Co., ST. Louis, MO, USA). MICs were determined by broth microdilution and interpreted according to the European Committee on Antimicrobial Susceptibility Testing (EUCAST) clinical breakpoints (http://www.eucast.org/clinical_breakpoints/) (EUCAST, 2021). In the absence of EUCAST clinical breakpoints, those of the Clinical and Laboratory Standards Institute were applied (Clinical and Laboratory Standards Institute, 2021).

### Bactericidal Assays

*In vitro* time-kill experiments were performed in duplicate in 20 mL tubes containing Cation Adjusted Mueller-Hinton broth (CA-MHB) (Difco, Detroit, MI) using a starting *inoculum* of 10^5^-10^6^ CFU/mL with ceftobiprole (1X, 2X and 4X MIC). Bactericidal activity was defined as a ≥3 log_10_ decrease in bacterial count at 24h (White et al., 1996). Statistical analysis was performed using GraphPad Prism (Version 8.4.0). All experiments were performed in triplicate. Data were represented as mean ±SD of triplicate experiments.

### Gene Amplification and Sequence Analysis

All isolates were molecularly characterized for the *pbp*4 gene sequence in order to analyze possible mutations and verify their role in influencing BPR activity. *pbp*4 was amplified by PCR and the entire gene was double-strand sequenced using oligonucleotides specifically designed for this study (**Supplemental Table 1**). Sequencing was performed using the Dye Terminator DNA sequencing kit V1.1 (Applied Biosystems TM), followed by purification using the DyeEx 2.0 Spin Kit (Quiagen, Hilden, Germany). The sequences obtained were corrected and analyzed using the Chromas Lite 2.1 program and then exported in FASTA format. Sequence alignment and gene and translated protein analysis were performed by using BLAST tool (Basic Local Alignment Search Tool) (https://blast.ncbi.nlm.nih.gov/Blast.cgi), CLC Sequence Viewer 8.0 and UniProt (www.uniprot.org). *E. faecalis* ATCC 47077, whose complete genome sequence is deposited at NCBI under the accession number CP025020.1., was used as reference.

### Real-Time Quantitative PCR

For real-time quantitative PCR (RT-qPCR) studies, 7 mL of bacterial suspensions (10^5^ CFU/mL) were incubated at 37°C until late-log-phase (0.1 OD_600_ ≅ 1x10^8^ CFU/mL); total RNA was extracted using the RNeasy^®^ Mini kit (Qiagen, Hilden, Germany), purified from contaminating DNA genomics and retro-transcripted in cDNA using the QuantiNova™ Reverse Transcription Kit (Qiagen, Hilden, Germany), according to the manufacturer’s instructions. cDNA was quantified using the Qubit™ 4 fluorometer.

RT-qPCR was performed in a Rotor-Gene Q (Qiagen, Hilden, Germany) instrument, using the QuantiNova™ SYBR^®^ Green PCR kit (Qiagen, Hilden, Germany), according to the manufacturer’s instructions. *pbp*4 (5RT) and 16S rRNA qPCR oligonucleotides were specifically designed for this study (**Supplemental Table 1**). For each sample, three biological replicates were prepared. Relative gene expression levels of transcription were calculated by the quantification cycle (Cq) method and normalized to the expression of 16S rRNA. Relative expression was calculated using the 2^- ΔΔ Ct^ method (Livak and Schmittgen, 2001). The data obtained were expressed as the fold-change in expression compared to that of the ATCC 47077 reference. Comparison of the expression levels of transcription of all strains and statistical analysis were conducted using the Relative Expression Software Tool “REST 2009” (Qiagen, Hilden, Germany) and GraphPad Prism (Version 8.4.0).

## Results

### *In Vitro* Antibacterial Activity

**Table 1** shows the susceptibility values of the 7 *E. faecalis* strains in study to β-lactams and comparator drugs. Five strains were found to be penicillin-resistant ampicillin-susceptible (PRAS), besides showing reduced susceptibility to ceftaroline (MICs ≥4 mg/L). Four out of 5 strains showed higher ceftobiprole MIC values (≥4 mg/L) and were reported as ceftobiprole non-susceptible (BPR-NS). All isolates were also susceptible to the other β-lactams tested (amoxicillin and imipenem), and susceptible to daptomycin, linezolid and tigecycline. High-level resistance to gentamicin (HLGR) (n=1), streptomycin (HLSR) (n=1) and both aminoglycosides (HLAR) (n=4) was detected. Vancomycin and teicoplanin resistance (VRE) was detected in 2 isolates and further found to be associated with the presence of the *van*A gene.

**Table 1:** Beta-lactams and comparator antimicrobial MIC values against *E. faecalis* clinical isolates.

Code	MIC values (mg/L)												
	P^1^	AMP	AML	IMI	BPR^2^	CPT^2^	VA	TEC	CN	S	LNZ	TGC	DAP^1^
**Efs1**	16	1	0.5	4	16	>256	0.5	2	>1024	>1024	4	0.25	0.5
**Efs2**	4	0.5	0.5	4	2	1	4	2	32	256	4	0.125	0.5
**Efs7**	64	4	4	4	8	>256	1	2	>1024	>1024	4	0.06	0.5
**Efs8**	16	4	1	2	4	32	>256	>256	>1024	128	4	0.125	0.5
**Efs11**	16	4	1	4	2	32	>256	128	>1024	>1024	2	0.25	0.5
**Efs18**	16	2	1	2	4	4	1	2	>2048	>1024	2	0.125	0.5
**Efs20**	4	2	0.5	1	0.25	0.5	1	0.5	32	>1024	2	0.06	1

^1^Penicillin and Daptomycin susceptibility values were established according to CLSI breakpoints (EUCAST breakpoints absent). ^2^Ceftobiprole and Ceftaroline: No EUCAST and CLSI official breakpoints; eCOFFs not determined.

P, Penicillin; AMP, Ampicillin; AML, Amoxicillin; IMI, Imipenem; BPR, Ceftobiprole; CPT, Ceftaroline; VA, Vancomycin; TEC, Teicoplanin; CN, Gentamicin; S, Streptomycin; LNZ, Linezolid; TGC, Tigecyclin; DAP, Daptomycin.

### Bactericidal Activity of Ceftobiprole

**Figure 1:**
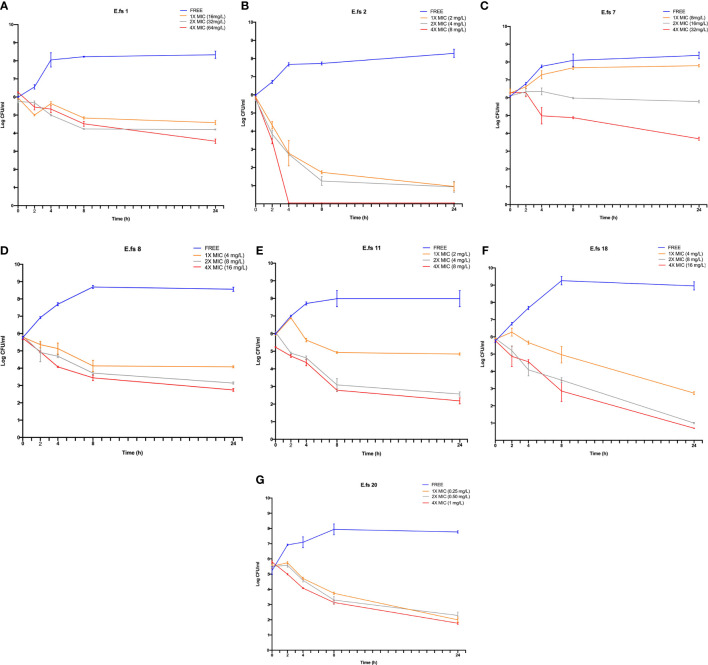
Time-kill assays of ceftobiprole (BPR) against the seven *E. faecalis* clinical isolates in study. Cell count was reported as log_10_ (CFU/ml) at 0, 2, 4, 8 and 24h Time-points (T0, T2, T4, T24); Ceftobiprole (BPR) exposure was tested at 1X, 2X and 4X MICs. Error bars represent standard deviations (±SD) of the mean of triplicate experiments **(A–G)**.

In BPR-NS, BPR exposure resulted in a potent bactericidal activity (3 to 5 log_10_) at 4X MIC after 24h. BPR-S strains showed a greater log reduction (3 to 5 log_10_) even at lower concentrations (i.e., 1X and/or 2X MIC). Enhanced killing activity was also observed at 8 h (**Figure 1**).

### *pbp4* Sequence Analysis and Significant Protein Alterations

**Figure 2:**
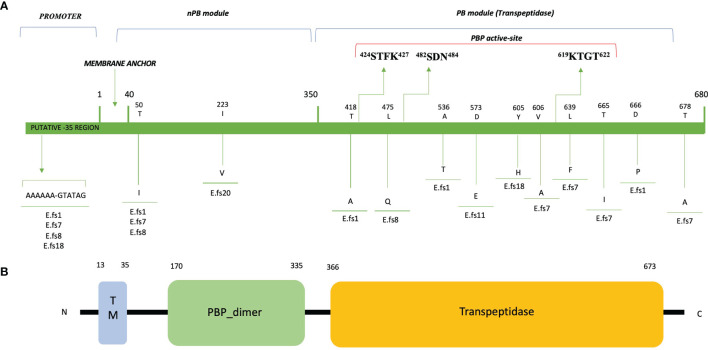
Amino acid substitutions and domain architecture of PBP4 of *E faecalis* clinical isolates. GenBank accession no. from OM032878 to OM032884. The numbers above the diagrams indicate residue numbers of the domain boundaries in the proteins. **(A)** Non-Penicillin Binding module (nPB); Penicillin Binding module (PB); TM, transmembrane helix region. ^424^STFK^427^, ^482^SDN^484^ and ^619^KTGT^622^: catalytic-site motifs (STFK_424_ includes the catalytic serine S_424_). **(B)** The domain architecture of PBP4 of *E faecalis* ATCC 47077 (OG1RF) was analyzed using SMART (Simple Modular Architecture Research Tool; http://smart.embl-heidelberg.de/) and reported in a simplified version.

PCR and sequence analysis of the *pbp*4 gene revealed some mutations that may account for the changes in PBP4 affinity and MIC increase in the 4 BPR-NS strains (MICs 4-16 mg/L). The adenine deletion (*del*A) in the promoter region, 8bp upstream of the -35 *consensus* site, was the carried by all 4 strains showing non-susceptibility to BPR and high-level resistance to penicillin (**Tables 1**, **2**). Sequence analysis of translated PBP4 identified 12 different missense mutations, 7 of which were next to the PBP catalytic sites (**Table 2**; **Figure 2**). Notably, the T418A mutation (Efs1) was located 6 amino acids (aa) upstream of the catalytic serine included in the ^424^STFK^427^ *motif* I; L475Q (Efs8), 7 aa upstream of the ^482^SDN^484^ *motif* II, involved in β-lactam leaving group protonation; the novel Y605H (Efs18) and V606A (Efs7), 13/14 aa upstream of the ^619^KTGT^622^ *motif* III, which facilitates substrate binding. Other mutations were found far from the PBP catalytic site: i) T50I, quite common in our sample (3/7), and I223V, only present in a fully susceptible strain (Efs20) in which it does not affect MICs to β-lactams, both located in the N-terminal end; ii) L639F, T665I and T678A (all in Efs7), and D666P (Efs1), located in the C-terminal end.

**Table 2:** Ceftobiprole MIC values (mg/L) and expression levels for *E. faecalis* clinical isolates, compared to sequence alterations.

Code	Phenotype characteristics	BPR MIC (mg/L)*^a^*	^§^Fold-change mean	Deletion in promoter region^b^	Amino acid substitutions in PBP4^d^											
							*PBP active-sites*									
					**_50_T**	**_223_I**	**_418_T**	**_475_L**	**_536_A**	**_573_D**	**_605_Y**	**_606_V**	**_639_L**	**_665_T**	**_666_D**	**_678_T**
**Efs20**	**PSAS; BPR-S; HLSR**	0.25	88. 80	–	**-**	**V**	**-**	**-**	**-**	**-**	**-**	**-**	**-**	**-**	**-**	**-**
**Efs2**	**PSAS; BPR-S; fully susceptible**	2	77.36	–	**-**	**-**	**-**	**-**	**-**	**-**	**-**	**-**	**-**	**-**	**-**	**-**
**Efs11**	**PRAS; BPR-S; VRE/*van*A; HLAR**	2	695.413	–	**-**	**-**	**-**	**-**	**-**	**E**	**-**	**-**	**-**	**-**	**-**	**-**
**Efs8**	**PRAS; BPR-NS; VRE/*van*A; HLGR**	4	4851.96	2013028_2013029 *del*A ***^c^***	**I**	**-**	**-**	**Q**	**-**	**-**	**-**	**-**	**-**	**-**	**-**	**-**
**Efs18**	**PRAS; BPR-NS; HLAR**	4	422.88	2013028_2013029 *del*A ***^c^***	**-**	**-**	**-**	**-**	**-**	**-**	**H**	**-**	**-**	**-**	**-**	**-**
**Efs7**	**PRAS; BPR-NS; HLAR**	8	571.068	2013028_2013029 *del*A ***^c^***	**I**	**-**	**-**	**-**	**-**	**-**	**-**	**A**	**F**	**I**	**-**	**A**
**Efs1**	**PRAS; BPR-NS; HLAR**	16	698.895	2013028_2013029 *del*A ***^c^***	**I**	**-**	**A**	**-**	**T**	**-**	**-**	**-**	**-**	**-**	**P**	**-**

^a^BPR, Ceftobiprole; ^b^a single base pair deletion 8 bases upstream of the putative -35 region; ^c^Accession number GenBank: CP025020.1 (ATCC47077); ^d^Protein ID GenBank: AEA94594.1 (ATCC47077); ^§^Fold-change expression levels relative to that of ATCC47077. Average of three independent experiments. HLSR, High Level Streptomycin Resistance; VRE, Vancomycin Resistant E. faecalis; HLAR, High Level Aminoglycosides Resistance; PRAS, Penicillin-Resistant Ampicillin-Susceptible; BPR-NS, Ceftobiprole Non-Susceptible; PSAS, Penicillin-Susceptible Ampicillin-Susceptible; BPR-S, Ceftobiprole Susceptible; HLGR, High Level Gentamicin Resistance. GenBank accession no. from OM032878 to OM032884.

Two further mutations in the region between the ^482^SDN^484^ and ^619^KTGT^622^ catalytic sites were detected (A536T; D573E) in Efs1 and Efs11, respectively; in Efs11, the D573E substitution potentially affects the MIC values of penicillin and ceftaroline (16 and 32 mg/L, respectively), but not those of ceftobiprole (2 mg/L).

Nucleotide Sequence Accession Numbers. The complete sequences of the pbp4 gene variants have been deposited in GenBank under accession numbers from OM032878 to OM032884.

### Increase in the Level of *pbp*4 Expression

The evaluation of *pbp*4 gene expression relative to that of the ATCC47077 (OG1RF) reference strain showed varying levels of upregulation in all strains, linked to their β-lactam MIC values. The PRAS/BPR-S Efs2 and Efs20 strains exhibited lower expression levels (≤10^2^ fold-change increase). All PRAS-BPR-NS strains carrying the *del*A in the *pbp*4 promotor region and mutations in the PBP catalytic sites displayed *pbp*4 overexpression with greater fold-change increases (0.5-4 x10^3^).

**Figure 3:**
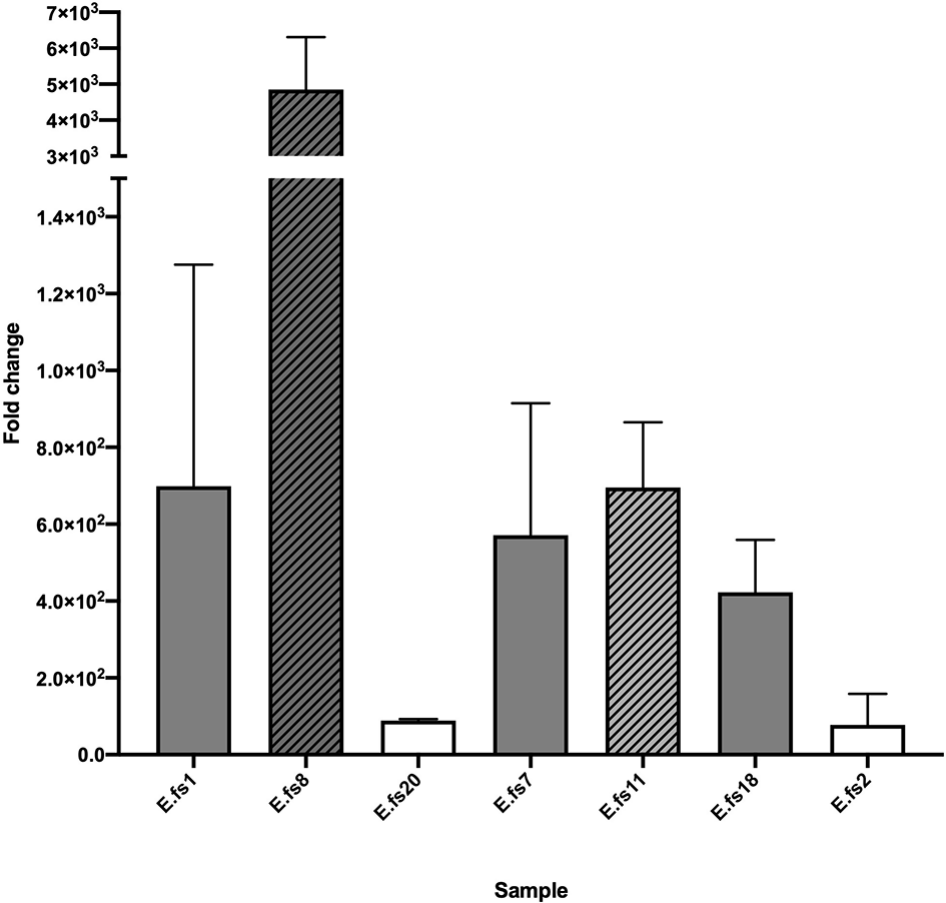
*pbp*4 gene expression analysis of the *E. faecalis* isolates in study, compared to that of the ATCC 47077 (OG1RF) reference. Evaluation of *pbp*4 relative mRNA expression expressed as fold-change (linear). 16S rRNA gene expression levels were used as calibrator. ATCC 47077 (OG1RF) was used as reference. Error bars indicate the average of three biological replicates in three RT-qPCR assays ± SD; PSAS, white; PRAS, gray. VRE/*van*A, pattern-filled bars.

We observed that *pbp*4 gene expression was more evident in the VRE/*van*A strains (PRAS/BPR-NS Efs8 and PRSA/BPR-S Efs11), with or without *del*A in the promoter region (**Figure 3**, pattern-filled bars).

### Correlation Between Sequence Alterations and Expression Level Increase

Missense mutations in the *pbp*4 sequence were common in BPR-NS and fully susceptible strains.

We observed a strong association between the increase in the level of *pbp*4 expression and the adenine deletion in the promoter region, upstream of the coding sequence. *del*A leads to a 3 log_10_ increase compared to that of the ATCC47077 (OG1RF) reference in all BPR-NS strains, demonstrating its role in establishing *in vitro* non-susceptibility to BPR, although it does not influence bactericidal activity (**Figure 3**).

The only exception was Efs11 (PRAS; CPT-NS; BPR-S), carrying only the D573E substitution. We were not able to demonstrate whether this mutation within the SDN/KTG catalytic sites alone may potentially interfere with the expression of *pbp*4 transcription, or if the higher upregulation may be related to the VRE/*van*A phenotype, as stated above. Further experiments are needed to confirm its role.

## Discussion

High resistance to penicillins in *E. faecalis* strains is rare, it can emerge after prolonged β-lactam therapy treatments (Rice et al., 2018). Nevertheless, the PRAS phenotype was described in several countries (Metzidie et al., 2006; Guardabassi et al., 2010; Conceição et al., 2014), but its epidemiological and clinical impact remains ambiguous, as ampicillin is the treatment of choice for enterococcal infections (Kristich et al., 2014a) and penicillin MIC values were never reported (Mendes et al., 2016). Recently, Kim et al. reported significative differences in mortality rates in patients with a PRAS *E. faecalis* BSI, likely due to the treatment failures of ampicillin and/or piperacillin (Kim et al., 2019).

PBP4, like other PBPs of the same class B (i.e., PBP2a of methicillin-resistant *S. aureus*) performs the cross-linking reaction of the peptidoglycan (PG) biosynthesis, but retains low responsiveness to beta-lactams. Due to its low-reactivity, PBP4 is considered the key basis for intrinsic resistance to cephalosporins in *E. faecalis* (Arbeloa et al., 2004).

Ceftobiprole, a novel cephalosporin that inhibits the PG cross-linking reaction by acylating the active-site serine of PBPs, maintains a higher affinity for PBP4. Due to its unique ability to target low-affinity PBP4, ceftobiprole is the best candidate as a valid therapeutic option for remarkable *E. faecalis* MDR-phenotypes such as PRAS and VRE. Alterations in this enzyme cause conformational changes that impact the structure of the catalytic *motifs* (Moon et al., 2018).

This study addressed the mechanism of non-susceptibility to ceftobiprole and the resulting interactions with *E. faecalis* clinical strains, all isolated from bloodstream infections.

A link between benzyl-penicillin resistance and 5^th^ generation cephalosporins non-susceptibility was observed. Our data suggest that this common insensitivity in PRAS isolates results from the involvement of *pbp*4 mutations in increased gene expression levels and alteration of the penicillin binding domain that could remodel the PBP/β-lactam complex.

Our *in vitro* dynamic data by time-kill curve assays showed that BPR exerts a bactericidal activity against *E. faecalis* isolates despite their MDR phenotypes (VRE, PRAS and BPR-NS), and PBP4 alterations, even after 8h, consistently with other studies (Werth and Abbott, 2015; Arias et al., 2007). After 24 hours, the bactericidal activity against all isolates - with or without significative PBP4 changes - was similar, suggesting that ceftobiprole maintains high affinity also for other PBPs.

The majority of the PBP4 mutations found in this study were already reported in the literature, frequently related to PRAS strains (Conceição et al., 2014; Infante et al., 2016; Gawryszewska et al., 2021), but the mechanisms underlying this relationship were not elucidated.

It is well known that the upstream region *consensus* sequence, in the bacterial promoters, can have an impact on the expression of downstream coding genes (Estrem et al., 1998). In *E. faecalis*, the involvement of an adenine deletion (*del*A) upstream of the -35 region of the *pbp*4 promoter was recently analyzed in a single strain (Rice et al., 2018). Supported by these observations, we provided a comprehensive analysis on a larger sample of clinical strains belonging to different antibiotic-resistance profiles.

In all BPR-NS, we observed that the *del*A upstream of the coding sequence results in *pbp*4 overexpression, hypothetically altering the binding of regulatory proteins. Elevated expression levels may cause increased transpeptidation, resulting in a highly cross-linked peptidoglycan. This demonstrates its role in establishing *in vitro* non-susceptibility to BPR without affecting its *cidal* activity.

The combination of *del*A with additional significant amino acid changes within the PBP4 active sites might result in destabilization and formation of a less competent β-lactam binding-complex. In one PRAS/BPR-NS strain (Efs1), the T418A mutation located 6 amino acids upstream of the catalytic serine included in the ^424^STFK^427^ *motif* I affects the MIC value of BPR (BPR 16 mg/L), which only remains bactericidal at the highest BPR concentration tested (4X MIC). On the contrary, the I223V mutation located in the N-terminal end, carried by a PSAS/BPR-S strain (Efs20), does not affect the MIC of β-lactams. This region is known to have no enzymatic function (Infante et al., 2016; Rice et al., 2018; Djorić et al., 2020), and this corroborates the excellent *in vitro* antibacterial and bactericidal activity of BPR against this strain, exerted at 1X, 2X and 4X MICs.

We observed that *pbp*4 was more overexpressed in the VRE/*van*A strains regardless of *del*A in the promoter region (Efs8 and Efs11) (**Figure 3**, pattern-filled bars); these strains also reported lower BPR MIC values (2-4 mg/L). This phenomenon was difficult to explain. In VRE/*van*A isolates, the DAla-DLac PG precursor is not processed by PBP4, as previously reported in *E. faecium* for PBP5 (al-Obeid et al., 1992). We could hypothesize that PBP4 may not work with the modified precursor ending in DLac as it may not be able to identify the target, and this may result in overexpression and subsequent buildup. Besides, production of precursors ending in DAla or in DLac alternatively responsible for resistance to cephalosporins or glycopeptides may promote enhanced cephalosporin susceptibility in the presence of vancomycin/beta-lactam association (Kristich et al., 2014b).

Even though the aim of this study was not to trace *E. faecalis* epidemiology, we detected three strains carrying PBP4 variants already reported in hospital-associated PRAS strains, epidemiologically related to the High-Risk Enterococcal Clonal Complex (HiRECC) CC87 (Kuch et al., 2012; Gawryszewska et al., 2021). In particular, Efs7 exhibited 4 combined mutations in PBP4 (designating the F3 variant), hypothetically responsible for its indifference to 1X MIC ceftobiprole concentration, in time-kill assays; Efs1, 3 combined mutations (E1 variant); and Efs8, a single mutation (C4 variant). This observation has a clinical and epidemiological impact: the evolution of nosocomial CCs is driven by the acquisition of resistance genes, and the spread of PBP4 variants, responsible for resistance to all beta-lactams, may potentially compromise the clinical efficacy of *E. faecalis* therapy in hospital settings.

In conclusion, in this study we revealed that benzyl-penicillin and 5^th^ generation cephalosporins interact with PBP4 in similar ways. In PRAS/BPR-NS *E. faecalis* clinical isolates, the interaction between increased *pbp*4 gene expression, due to the *del*A in the upstream region *consensus* sequence, and the supposed remodeling of the penicillin-binding domain, due to alterations in the PBP4 amino acid sequence, influence their β-lactams susceptibility profiles without affecting BPR *cidal* activity.

In the light of the above, we recommend penicillin MICs determination not just for clinical, but also and foremost for epidemiological purposes, to evaluate the spread of isolates belonging this difficult-to-treat epidemic PRAS phenotype as well as to address the proper antimicrobial treatment options for these infections.

The major limitation of this study is the lack of a functional evaluation of *del*A and amino acid substitutions in PBP4. Further experimental approaches such as whole genome sequence analysis and site-directed mutagenesis, should be attempted to confirm the genetic basis of altered beta-lactams/PBP4 complexes induced by sequence substitutions.

## Data Availability Statement

The data presented in the study are deposited in the GenBank repository (https://www.ncbi.nlm.nih.gov/genbank), under accession numbers from OM032878 to OM032884.

## Author Contributions

FC designed the research and directed the project. LL, MC, and FC conducted the research. LL, MC and FC analyzed the data. LL, MC, SS, and FC wrote the manuscript. FC and SS, edited and reviewed the manuscript. All authors read and approved the manuscript.

## Funding

This study was partially supported by ADVANZ PHARMA and Programma PIACERI [CovDock]-Linea di intervento 2’, University of Catania, Dept. of Biomedical and Biotechnological Sciences (BIOMETEC).

## Conflict of Interest

The authors declare that the research was conducted in the absence of any commercial or financial relationships that could be construed as a potential conflict of interest.

## Publisher’s Note

All claims expressed in this article are solely those of the authors and do not necessarily represent those of their affiliated organizations, or those of the publisher, the editors and the reviewers. Any product that may be evaluated in this article, or claim that may be made by its manufacturer, is not guaranteed or endorsed by the publisher.
