# Supplementary material for: Impact of PBP4 Alterations on β-Lactam Resistance and Ceftobiprole Non-Susceptibility Among Enterococcus faecalis Clinical Isolates
Source: Front Cell Infect Microbiol. 2022 Jan 20;11:816657. doi: 10.3389/fcimb.2021.816657 (PMC8811369; doi:10.3389/fcimb.2021.816657)
Supplement: Supplementary file 1 [file Table_1.docx]

**Supplemental Table S1**

**(A) Oligonucleotides designed in this study for pbp4 gene sequencing.**

| Primer | Sequence (5-3) | PCR product size | Tm |
| --- | --- | --- | --- |
| pbp4-up | GTGTCCCCCATATTATCAGGTTC | 2429bp | 60.6 °C |
| pbp4-dw | CGCTTCATTGTAGCACACTTTCC |  | 60.6 °C |
| pbp4-up | GTGTCCCCCATATTATCAGGTTC | 822bp | 60.6 °C |
| pbp4.1-dw | GTTTGGCATTATCTACTTGAATCG |  | 57.6°C |
| pbp4.1-up | GTCTTATCAATCAAGTATCGCCAA | 747bp | 57.6°C |
| pbp4.2-dw | CTAACCGTTGCTAATAAATCGCC |  | 58.9°C |
| pbp4.3-up | AAATTAACGATTGACAGTGGCGT | 511bp | 57.1 °C |
| pbp4.3-dw | TTGGTAAATCTAGTTCCTCACCGA |  | 59.3°C |
| pbp4.2-up | CAAGTCCAGTCAATCTGCGTACC | 722bp | 62.4 °C |
| pbp4-dw | CGCTTCATTGTAGCACACTTTCC |  | 60.6°C |

**(B) Oligonucleotides designed in this study to evaluate gene expression by RT-qPCR.**

| Primer | Sequence (5-3) | PCR product size | Tm |
| --- | --- | --- | --- |
| E.fs 16S UP | CGTCAAATCATCATGCCCC | 299 bp | 50.1 °C |
| E.fs 16S DW | CCCCAATCATCTATCCCACC |  | 51.2 °C |
| E.fs 5RT pbp4 UP | TTTGACGAAGTGGGCGTAG | 132 bp | 52.1 °C |
| E.fs 5RT pbp4 DW | GGACCCATCCTTGGCTTAAC |  | 52.4 °C |
