# Supplementary material for: Impact of PBP4 Alterations on β-Lactam Resistance and Ceftobiprole Non-Susceptibility Among Enterococcus faecalis Clinical Isolates
Source: Front Cell Infect Microbiol. 2022 Jan 20;11:816657. doi: 10.3389/fcimb.2021.816657 (PMC8811369; doi:10.3389/fcimb.2021.816657)
Supplement: Supplementary file 2 [file Table_2.docx]

**Supplemental Table S2. RTq-PCR raw data obtained by an Excel spreadsheet and the** **Relative Expression Software Tool “REST 2009” REST software. Fold-change values were calculated using the 2^-ΔΔCt^ method. Each sample was compared with ATCC 47077 reference, arbitrarily set as 1.**

| Gene | Type | Reaction Efficiency | ∆Ct SAMPLE | ∆Ct ATCC 47077 | ∆∆Ct | Fold change expression | Std. Error | 95% C.I. | P(H1) | Result |
| --- | --- | --- | --- | --- | --- | --- | --- | --- | --- | --- |
| E.fs1 |  |  |  |  |  |  |  |  |  |  |
| 16S | REF | 1 |  |  |  | 1 |  |  |  |  |
| PBP4 (5RT) | TRG | 1 | 2,43 | 11,28666667 | -8,856666667 | 463,577 | 350,930-603,917 | 299,390-753,838 | 0,033 | UP |
| E.fs1 |  |  |  |  |  |  |  |  |  |  |
| 16S | REF | 1 |  |  |  | 1 |  |  |  |  |
| PBP4 (5RT) | TRG | 1 | 1,315 | 11,72 | -10,405 | 1.355,87 | 1.233,647-1.491,339 | 1.206,119-1.524,533 | 0 | UP |
| E.fs1 |  |  |  |  |  |  |  |  |  |  |
| 16S | REF | 1 |  |  |  | 1 |  |  |  |  |
| PBP4 (5RT) | TRG | 1 | 2,485 | 10,6 | -8,115 | 277,242 | 214,272 - 368,298 | 186,248 - 415,757 | 0 | UP |
| E.fs8 |  |  |  |  |  |  |  |  |  |  |
| 16S | REF | 1 |  |  |  | 1 |  |  |  |  |
| PBP4 (5RT) | TRG | 1 | -0,343333333 | 11,28666667 | -11,63 | 3.169,41 | 2.483,169-4.191,943 | 2.028,059-4.662,168 | 0 | UP |
| E.fs8 |  |  |  |  |  |  |  |  |  |  |
| 16S | REF | 1 |  |  |  | 1 |  |  |  |  |
| PBP4 (5RT) | TRG | 1 | -1,865 | 10,6 | -12,465 | 5653,78 | 4.102,502 - 8.343,652 | 3.243,084 - 10.050,615 | 0 | UP |
| E.fs8 |  |  |  |  |  |  |  |  |  |  |
| 16S | REF | 1 |  |  |  | 1 |  |  |  |  |
| PBP4 (5RT) | TRG | 1 | -1,865 | 10,626 | -12,48 | 5732,704 | 2.990,599 - 12.331,455 | 2.184,285 - 15.556,710 | 0 | UP |
| E. FS20 |  |  |  |  |  |  |  |  |  |  |
| 16S | REF | 1 |  |  |  | 1 |  |  |  |  |
| PBP4 (5RT) 5RT | TRG | 1 | 4,886666667 | 11,28666667 | -6,4 | 84,449 | 66,455 - 112,501 | 54,037 - 123,157 | 0,03 | UP |
| E. FS20 |  |  |  |  |  |  |  |  |  |  |
| 16S | REF | 1 |  |  |  | 1 |  |  |  |  |
| PBP4 (5RT) | TRG | 1 | 4,105 | 10,6 | -6,495 | 90,197 | 69,262 - 118,081 | 65,202 - 124,955 | 0 | UP |
| E. FS20 |  |  |  |  |  |  |  |  |  |  |
| 16S | REF | 1 |  |  |  | 1 |  |  |  |  |
| PBP4 (5RT) | TRG | 1 | 4,105 | 10,626 | -6,52 | 91,773 | 64,624 - 131,019 | 60,836 - 138,647 | 0 | UP |
|  |  |  |  |  |  |  |  |  |  |  |
|  |  |  |  |  |  |  |  |  |  |  |
| E.fs7 |  |  |  |  |  |  |  |  |  |  |
| 16S | REF | 1 |  |  |  | 1 |  |  |  |  |
| PBP4 (5RT) | TRG | 1 | 1,52 | 8,966666667 | -7,446666667 | 174,45 | 145,086 - 225,972 | 142,025 - 240,295 | 0 | UP |
| E.fs7 |  |  |  |  |  |  |  |  |  |  |
| 16S | REF | 1 |  |  |  | 1 |  |  |  |  |
| PBP4 (5RT) | TRG | 1 | 1,025 | 10,6 | -9,575 | 762,715 | 585,489 - 996,269 | 560,981 - 1.037,773 | 0 | UP |
| E.fs7 |  |  |  |  |  |  |  |  |  |  |
| 16S | REF | 1 |  |  |  | 1 |  |  |  |  |
| PBP4 (5RT) | TRG | 1 | 1,025 | 10,626 | -9,6 | 776,047 | 220,326 - 2.740,819 | 211,104 - 2.855,001 | 0 | UP |
| E. FS11 |  |  |  |  |  |  |  |  |  |  |
| 16S | REF | 1 |  |  |  | 1 |  |  |  |  |
| PBP4 (5RT) | TRG | 1 | -0,833333333 | 8,966666667 | -9,8 | 891,44 | 657,460-1278,290 | 643,591-1359,312 | 0 | UP |
| E. FS11 |  |  |  |  |  |  |  |  |  |  |
| 16S | REF | 1 |  |  |  | 1 |  |  |  |  |
| PBP4 (5RT) | TRG | 1 | 1,39 | 10,6 | -9,21 | 592,224 | 455,047 - 777,016 | 422,033 - 832,926 | 0 | UP |
| E.fs11 |  |  |  |  |  |  |  |  |  |  |
| 16S | REF | 1 |  |  |  | 1 |  |  |  |  |
| PBP4 (5RT) | TRG | 1 | 1,39 | 10,626 | -9,235 | 602,576 | 508,419 - 719,974 | 471,532 - 771,779 | 0 | UP |
| E. FS18 |  |  |  |  |  |  |  |  |  |  |
| 16S | REF | 1 |  |  |  | 1 |  |  |  |  |
| PBP4 (5RT) | TRG | 1 | 2,54 | 11,72 | -9,18 | 580,037 | 563,870-596,781 | 557,581-603,428 | 0 | UP |
| E. FS18 |  |  |  |  |  |  |  |  |  |  |
| 16S | REF | 1 |  |  |  | 1 |  |  |  |  |
| PBP4 (5RT) | TRG | 1 | 2,185 | 10,6 | -8,415 | 341,324 | 266,563 - 460,996 | 217,155 - 544,668 | 0 | UP |
| E. FS18 |  |  |  |  |  |  |  |  |  |  |
| 16S | REF | 1 |  |  |  | 1 |  |  |  |  |
| PBP4 (5RT) | TRG | 1 | 2,185 | 10,626 | -8,44 | 347,291 | 250,442 - 507,974 | 204,022 - 600,172 | 0 | UP |
| E.fs2 |  |  |  |  |  |  |  |  |  |  |
| 16S | REF | 1 |  |  |  | 1 |  |  |  |  |
| PBP4 (5RT) | TRG | 1 | 4,305 | 11,72 | -7,415 | 170,662 | 162,998-178,824 | 159,361-182,804 | 0 | UP |
| E.fs2 |  |  |  |  |  |  |  |  |  |  |
| 16S | REF | 1 |  |  |  | 1 |  |  |  |  |
| PBP4 (5RT) | TRG | 1 | 5,67 | 10,6 | -4,93 | 30,484 | 23,590 - 40,586 | 20,310 - 46,140 | 0 | UP |
| E.fs2 |  |  |  |  |  |  |  |  |  |  |
| 16S | REF | 1 |  |  |  | 1 |  |  |  |  |
| PBP4 (5RT) | TRG | 1 | 5,67 | 10,626 | -4,952 | 30,953 | 30,953 - 30,953 | 30,953 - 30,953 | 0 | UP |
